# Editorial: Network-Oriented Approaches to Anticancer Drug Response

**DOI:** 10.3389/fbioe.2021.692369

**Published:** 2021-05-24

**Authors:** Paola Lecca, Juan Manuel Corchado, Daniela Besozzi

**Affiliations:** ^1^Faculty of Computer Science, Free University of Bozen-Bolzano, Bolzano, Italy; ^2^Department of Computer Science and Automation, University of Salamanca, Salamanca, Spain; ^3^Department of Informatics, Systems and Communication, University of Milano-Bicocca, Milan, Italy

**Keywords:** network biology, cancer biology, control theory, genome biology, pharmacokinetics, pharmacodynamics, drug response and resistance

Variational calculus and the theory of control, one of its most familiar branches in the applied sciences, have been used in modeling and analysis of biological systems and in biotechnology for several years. The work by Norbert Wiener on cybernetics in 1965, the work by Walter Cannon on homeostasis in 1929 (Cannon, 1929), and the early work by Claude Bernard - indicated as the first systems biologist (Noble, 2007) - on the *milieu interieur* in 1865 (Bernard, 1984), are the first evidence of an increasingly fruitful interaction between control theory and life sciences (Azar, 2020). The best known results of this interaction have been in the field of biomedical engineering, but, we are now seeing an increasing use of control theory methods in areas such as pharmacology, medicine, and molecular biology. The application of control theories in these branches of life sciences has been made possible by the emergence and consolidation of *systems biology*, which has proposed a new scientific paradigm. This paradigm shifted the focus of biochemistry and molecular biology and their applicative domains, such as medicine and pharmacology, from the study of structural and functional properties of molecules, genes, proteins, functional complexes to the network of their interactions. Viewing a biological process as the temporal evolution of the network of interactions between the variables involved in this process has enabled the use of approaches based on systems theory and graph theory to analyse, model and simulate a biological system. Control theory naturally fits into the theoretical framework of network theory, and in this context it is known as network control theory. Network biology and its declinations as network genomics (Ideker, 2007; Charitou et al., 2016), network proteomics (Arrell and Terzic, 2012; Chisanga et al., 2016; Zhang et al., 2018), and network pharmacology (Hopkins, 2007, 2008; Casas et al., 2019) are all examples of the application of network theory approaches in life sciences.

The aim of this special issue was precisely to collect reviews and innovative research contributions in this new area at the intersection of network biology, control theory, medicine, and pharmacology. In particular, among the various application domains, we focused on cancer medicine and anticancer drug response. Predictive modeling of anticancer drug responses is a crucial step to speed up drug design while minimizing the risk of direct *in vivo* tests, and ultimately to accomplish the precision medicine in oncology. This is a non-trivial issue, and there is the urgency to improving the prediction performance by combining multiple types of genome-wide molecular data and by implementing tailored *wet* and *in silico* analyses.

In this context and with this objective in mind, the special issue presents seven articles, of which two are reviews and five are original research works concerning *in silico* and *in vitro/in vivo* analyses. In total, 34 authors contributed to the special issue. The articles are a collection of highly complementary contributions proposing advanced computational methods based on the integration and intelligent use of different types of data to exploit their predictive power. Here we briefly report the main contribution of each article to emphasize their complementarity aspects.

The article by Angaroni et al., “An Optimal Control Framework for the Automated Design of Personalized Cancer Treatments” is motivated by the effectiveness of control theory to therapy design and testing: the authors propose the Control Theory for Therapy Design (CT4TD) framework, which employs optimal control theory on patient-specific pharmacokinetics and pharmacodynamics models, to deliver optimized therapeutic strategies.

Differently from the work by Angaroni et al., the article by Cheng et al., "Identification of the Significant Genes Regulated by Estrogen Receptor in Estrogen Receptor-Positive Breast Cancer and Their Expression Pattern Changes When Tamoxifen or Fulvestrant Resistance Occurs" presents a data-driven study to identify the significant genes regulated by ER in ER-positive breast cancer. The authors explored the alteration of the pathways involving these genes when tamoxifen or fulvestrant resistance occurs. The study is therefore an exploration of currently available data to identify control and regulated genes in gene networks that may have been altered by pharmacological treatments. In addition to approaches based on a computational models and validated on datasets, the integration of data as well as computational approaches of different types is another proposal for the analysis of the controllability of complex systems. The article by Yuan et al., “Computational Prediction of Drug Responses in Cancer Cell Lines From Cancer Omics and Detection of Drug Effectiveness Related Methylation Site” is an *in silico*, data-driven study in which the authors propose a strongly integrative computational model to predict drug responses in cancers. They integrate data of cancer genomics, transcriptomics, epigenomics, and compound chemical properties. The model has been trained on pharmacogenomics data, to detect the methylation sites closely related to drug effectiveness.

In the field of cancer medicine, the theory of control and in particular the theory of optimal control can be used not only for the analysis of systems whose control variables are genes and proteins, but also for the analysis of a system whose control variables are drug treatments and the objective function is to determine which drug combinations are best in terms of efficacy and reduced side effects. On this line, Spolaor et al., in “Screening for combination cancer therapies with dynamic fuzzy modeling and multi-objective optimization” present a computational approach that allows to efficiently identify novel combination of cancer therapies. More importantly, the results reported in this study show that the method can suggest potential therapies consisting in a small number of molecular targets, and thus with limited side-effects.

In addition to the variety of computational approaches and biological systems presented in the articles mentioned so far, the special issue also includes an original Research Article in which the physical system considered is the cell-cell interaction network. This is the work by Belenchia et al., “Agent-based Learning Model for the Obesity Paradox in RCC,” in which the authors propose a multi-agent based model of tumor, based on the cell interactions network. The simulations of this model are used to generate hypothesis on the reason why immunotherapy treatment of Renal Cell Carcinoma better controls the tumor in obese patients than in lean patients.

The special issue could not fail to include reviews, in order to give the reader an accurate and up-to-date picture of the state of the art. The article by Frieboes et al., “Modeling of Nanotherapy Response as a Function of the Tumor Microenvironment: Focus on Liver Metastasis” is a review on the drug transport mechanisms through tumor microenvironment and the studies carried on with advanced *in vitro* 3D tissue models as well as with *in silico* mathematical modeling to optimize drug transport and uptake by cancer cells. Next to this contribution and to the contribution by Angaroni et al. and parallel to its content, the review “Control Theory and Cancer Chemotherapy: How They Interact” by Lecca, describes the mathematical foundation of control theory applied to pharmacokinetics and pharmacodynamics models.

## Author Contributions

All authors listed have made a substantial, direct and intellectual contribution to the work, and approved it for publication.

## Conflict of Interest

The authors declare that the research was conducted in the absence of any commercial or financial relationships that could be construed as a potential conflict of interest.
